# Barriers and enablers of health system adoption of kangaroo mother care: a systematic review of caregiver perspectives

**DOI:** 10.1186/s12887-016-0769-5

**Published:** 2017-01-25

**Authors:** Emily R. Smith, Ilana Bergelson, Stacie Constantian, Bina Valsangkar, Grace J. Chan

**Affiliations:** 1 Department of Global Health and Population, Harvard T. H. Chan School of Public Health, 665 Huntington Ave., Building 1, Boston, MA 02115 USA; 2 Division of Medicine Critical Care, Boston Children’s Hospital, Boston, MA USA; 3 Saving Newborn Lives, Save the Children, Washington, D.C., USA

**Keywords:** Kangaroo mother care, Skin to skin care, Health system integration, Mother, Father, Family, Caregiver

## Abstract

**Background:**

Despite improvements in child survival in the past four decades, an estimated 6.3 million children under the age of five die each year, and more than 40% of these deaths occur in the neonatal period. Interventions to reduce neonatal mortality are needed. Kangaroo mother care (KMC) is one such life-saving intervention; however it has not yet been fully integrated into health systems around the world. Utilizing a conceptual framework for integration of targeted health interventions into health systems, we hypothesize that caregivers play a critical role in the adoption, diffusion, and assimilation of KMC. The objective of this research was to identify barriers and enablers of implementation and scale up of KMC from caregivers’ perspective.

**Methods:**

We searched Pubmed, Embase, Web of Science, Scopus, and WHO regional databases using search terms ‘kangaroo mother care’ or ‘kangaroo care’ or ‘skin to skin care’. Studies published between January 1, 1960 and August 19, 2015 were included. To be eligible, published work had to be based on primary data collection regarding barriers or enablers of KMC implementation from the family perspective. Abstracted data were linked to the conceptual framework using a deductive approach, and themes were identified within each of the five framework areas using Nvivo software.

**Results:**

We identified a total of 2875 abstracts. After removing duplicates and ineligible studies, 98 were included in the analysis. The majority of publications were published within the past 5 years, had a sample size less than 50, and recruited participants from health facilities. Approximately one-third of the studies were conducted in the Americas, and 26.5% were conducted in Africa.

We identified four themes surrounding the interaction between families and the KMC intervention: buy in and bonding (i.e. benefits of KMC to mothers and infants and perceptions of bonding between mother and infant), social support (i.e. assistance from other people to perform KMC), sufficient time to perform KMC, and medical concerns about mother or newborn health. Furthermore, we identified barriers and enablers of KMC adoption by caregivers within the context of the health system regarding financing and service delivery. Embedded within the broad social context, barriers to KMC adoption by caregivers included adherence to traditional newborn practices, stigma surrounding having a preterm infant, and gender roles regarding childcare.

**Conclusion:**

Efforts to scale up and integrate KMC into health systems must reduce barriers in order to promote the uptake of the intervention by caregivers.

## Background

Despite improvements in child survival in the past four decades, an estimated 6.3 million children under the age of five die each year, and more than 40% of these deaths occur in the neonatal period [1]. Complications related to preterm birth is the leading cause of death among children under five [2]. Effective implementation, at scale, of evidence-based interventions to reduce complications of preterm birth and associated neonatal mortality is needed.

Kangaroo Mother Care (KMC) is one such evidence-based, life-saving intervention. There are four components of KMC including: 1) early, continuous, and prolonged skin-to-skin contact between infant and caregiver, 2) exclusive breastfeeding, 3) early discharge from hospital, and 4) adequate support for caregiver and infant at home [3, 4]. In addition to providing thermal control, KMC is associated with a 36% reduced risk of neonatal mortality among low birth weight newborns compared to conventional care, as well as a significantly reduced risk of sepsis, hypoglycemia, and hypothermia [5].

Despite the strong evidence regarding the improved health outcomes among preterm or low birth weight infants receiving KMC, including a recent recommendation by the World Health Organization that KMC should be routine care for newborns weighing less than 2000 g [6], this intervention has never been fully integrated into health systems around the world. A previous systematic review identified barriers to health system adoption of KMC and noted that families play an important role in KMC adoption [7]. Further, the review noted that family interactions with the health system were critical to KMC adoption. Caregivers (e.g. mothers, fathers, and families) are key implementers and beneficiaries of KMC. We explore the barriers and enablers of KMC implementation from the caregiver perspective in greater detail.

In order to understand the role of families in the adoption, diffusion, and assimilation of KMC, we build on a conceptual framework for integration of targeted health interventions into health systems [7, 8]. This framework promotes analysis in five areas including: (1) definition of the problem; (2) definition and attributes, such as the ‘relative advantage’ and ‘complexity’, of the intervention package; (3) the adoption system including key actors, their interest, values and the power dynamics between them; (4) health system characteristics; and (5) the broad context including demographic, economic, and cultural factors.

Using this framework, we analyzed how caregivers perceive the risks and benefits of the intervention, as well as their values and interests surrounding KMC. Specifically, we identified barriers and enablers of implementation and scale up of kangaroo mother care based on the first systematic review on KMC implementation and uptake from the caregiver perspective.

## Methods

In order to identify research studies for this review, we searched Pubmed, Embase, Web of Science, Scopus, AIM, LILACS, IMEMR, IMSEAR, and WPRIM. Search terms included: ‘kangaroo mother care,’ or ‘kangaroo care,’ or ‘skin to skin care’. Studies published between January 1, 1960 and August 19, 2015 were included. We also reviewed the references of published systematic reviews, searched unpublished programmatic reports, and requested data from the Saving Newborn Lives Program at Save the Children. We excluded studies if they did not include human subjects and primary data collection. To be eligible for inclusion into the review, published work had to include information about barriers to or enablers of successful implementation of KMC from the family perspective based on the experience of caregivers and health providers who had implemented KMC.

Two independent reviewers used a standardized data abstraction form to assess eligibility and abstract data from each article. If the case reviewers did not agree about the inclusion of a study, a third reviewer broke the tie. Each eligible study was assessed for the potential risk of bias in five domains including: selection bias, appropriateness of data collection, appropriateness of data analysis, generalizability, and consideration of ethics [9].

Through several iterations of manual annotation and indexing, two researchers coded themes, perspectives and experiences using NVivo software. Abstracted data were linked to the conceptual framework using a deductive approach, and themes were identified within each of the five framework areas. Narratives were constructed around each major theme, and we used quotes to summarize perspectives from each study. The major themes and narratives were used to develop matrices where we defined important concepts, the range and nature of each theme, and the relationship between themes. We used this meta-synthesis approach based on our objective of understanding caregiver perception and our hope that the results can inform policy and enhance our understanding of how to implement this complex intervention within health systems [10].

## Results

**Fig. 1:**
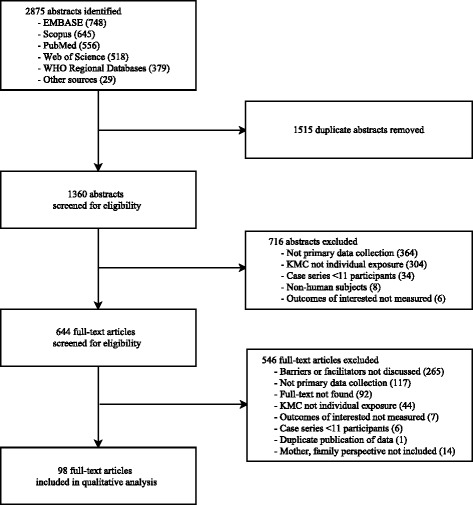
Systematic review flow chart

We identified a total of 2875 abstracts (748 in Embase, 645 in Scopus, 556 in Pubmed, 518 in Web of Science, 379 in WHO Regional Databases, and 29 from other sources). There were 1360 abstracts after removing duplicates. 716 were excluded after title and abstract review, and the full text was reviewed for 644. A total of 98 were included in the analysis (Fig. 1). All studies were considered of sufficient quality to include in the analysis.

The majority of publications were published within the past 5 years, had a sample size less than 50, and recruited participants from health facilities. One-third of the studies were conducted in the Americas, 26.5% in Africa, 16.3% in Europe, and the remaining in Southeast Asia, Eastern Mediterranean, Western Pacific, or in multiple regions. More than half of the studies were conducted in an area with a neonatal mortality rate < 15 deaths per 1000 live births (Table 1).

**Table 1:** Characteristics of included studies (*N* = 98)

Study Charactersitics	Percent
Year	
2010 to 2014	55.1
2000 to 2009	34.7
1988 to 1999	3.1
Missing	6.1
Sample Size	
< 50	53.1
50 to <1000	7.1
100 to <200	7.1
≥ 200	32.7
NMR (deaths per 1000 live birth)	
< 5	31.6
5 to <15	24.5
15 to <30	32.7
≥ 30	4.1
Missing	7.1
Setting (rural or urban)	
Urban	44.9
Urban and rural	10.2
Rural	6.1
Missing	38.8
Population source	
Health facility	60.2
NICU or stepdown unit	27.6
Community or population-based surveillance	11.2
Missing	1.0
Gestational Age	
Preterm 34 to <37 weeks	11.2
All gestational ages	11.2
Very preterm <34 weeks	7.1
Mixed preterm and very preterm <37 weeks	6.1
Full term ≥ 37 weeks	4.1
Missing	60.2
Birth weight	
Low birth weight 1500 to <2500 g	13.3
All birth weights	12.2
Mixed low and very low birth weight <2500 g	7.1
Very low birth weight <1500 g	4.1
Missing	63.3

After analyzing the data collected from the family perspective, we confirmed that caregivers are an essential component of the KMC adoption system as they are the primary decision-makers and are responsible for infant feeding and skin-to-skin contact. We identified four themes surrounding the interaction between caregivers and the KMC intervention: buy in and bonding, social support, time, and medical concerns. Furthermore, we identified barriers and enablers of KMC adoption by families within the context of the health system and the broader social context. These themes are summarized in Table 2.

**Table 2:** Matrix of enablers and barriers to KMC from the mother, father, and family perspective

	**The Intervention**				**Health System**	**Broad Context**
	**Buy-in and Bonding**	**Social Support**	**Time**	**Medical Concerns**	**Access to Care**	**Cultural Norms**
**Barriers**	**KMC felt forced** · Were unaware of the benefits of KMC · Were expected to perform KMC with little or no instruction · Could not see newborn during KMC · Did not feel a bond with the infant · Perceived newborn did not enjoy KMC	**Support from Society** · Fear, guilt doing KMC publically · Felt KMC was role of mother · Mothers did not want father to perform KMC	· Caregivers unable to devote time · Other responsibilities at home or work interfered · Mothers lonely and depressed in KMC ward · Time needed to commute from home to hospital was too much	**Mothers** · Fatigue · Postpartum depression · Pain hindered KMC, particularly after a C-section · Discomfort sleeping upright	**Financing** · Cost associated with travel, food, lodging, parking, clinical fees · Lack of transport and distance to facility	**Traditional Newborn Care** · Infants traditionally carried on back, thus carrying on the front seemed odd · Bathing practices interfered · If breast feeding not pursued KMC less likely to continue · Considered unclean where diapers not used **Gender Roles** **Stigma**
		**Support from HCWs** · Did not respect family privacy · Unsupportive, loud, uncaring			**Service Delivery** · Lack of privacy · Lack of necessary resources	
	**Stigma** · Mothers reported shame of having a preterm infant · Caregivers lied about carrying a newborn on their chest · Others presumed the newborn was ill or deformed	**Support from Family** · Mothers-in-law and grandmothers did not approve · Bad attitudes and peer pressure negatively influenced desire to perform KMC				
**Enablers**	**Benefits For Newborns** · Slept longer, less anxious, happier, more willing to feed	**Support from Society** · Societal acceptance of paternal involvement	· Parents preferred to practice KMC at home than at the facility to at tend to other responsibilities · Unlimited visitation hours at health facility	**Mothers** · KMC helped mother’s recover from post-partum depression · KMC helped to relieve stress and promote emotional well-being	**Financing** · Belief that KMC cut down hospital bills due to early discharge · Assumed to be a cheaper than incubator care · Parents more likely to stay if services were free **Service Delivery** · Private, quiet spaces for KMC	**Gender Roles** · Normalization of paternal involved in child care
	**Benefits For Caregivers** · KMC was calming, relaxing, comforting, natural, instinctive, secure, logical, healing · Created a family bond, inspired caregiver confidence · Sped emotional and physical recovery of mother · Made caregivers feel useful	**Support from HCWs** · Mothers less apprehensive to practice KMC. Best results with continuous training and support **Support from Family** · Grandmothers, sisters, others helping with chores increased uptake and duration of KMC · Paternal support crucial to success of KMC, they alleviate workload, support, encourage, increase mother’s confidence · More likely to understand and respond well if mother explained KMC				

### Barriers and enablers for caregiver adoption of KMC

#### Caregiver buy-in and bonding

Buy-in and bonding referred to the acceptance of KMC, belief in the benefits of KMC to mothers and preterm infants, and reported perceptions of bonding between mother and infant.

Uptake of KMC was impaired by limited buy-in to KMC by mothers, fathers, and families. For example: *“My experience told me this KMC was not right…so before caesarean section (in the meeting with a neonatal nurse). I was worried about it.”* (Father) [11]. Mothers were less likely to accept KMC if healthcare workers could not clearly explain the benefits of KMC. Parents reported that they were simply told to perform KMC without explanation why or how to do so, and the feeling that KMC was forced upon them hindered buy-in from caregivers [12]. Another barrier to parental buy-in occurred when caregivers perceived that their newborn did not enjoy KMC. In some areas due to the hot climate, parents observed their infant became irritable or “stinky” during SSC [13]. Less frequently, caregivers mentioned discomfort at not being able to see their newborn during KMC [14]. Another barrier was lack of bonding by mothers with their preterm infants [11]. In some cases lack of bonding with the infant was due to fear, stigma, shame, guilt, or anxiety about having a preterm infant [15, 16], and some did not want to keep the baby at all [17]. For example: “*I wished that I had had a miscarriage instead of delivering this preterm, it would be better. I never thought that this baby would survive; I thought that it would die any time”* (Mother) [17].

However, positive perceptions among mothers, fathers, and families regarding the potential benefits of the intervention promoted KMC uptake. Caregivers who successfully implemented KMC perceived that performing KMC calmed their baby [18–22]. These mothers observed their newborns sleeping longer during skin-to-skin contact; infants were described as less anxious, more restful, more willing to breastfeed, and happier to be in SSC position than in an incubator [18, 23]. KMC was also perceived as a healing mechanism for the parents. It helped mothers and fathers recover emotionally and physically, as well as create a family bond [24]. KMC made mothers feel useful. For example: *“Every time I hold her, the monitors-everything-did better. Her oxygen SATs did better, I really think I helped her…and think that the human contact and…hearing my heart and everything, I really think that helped her”* [19]. Some fathers reported feeling needed and enjoyed participating in the early care of their newborn. Further, families using KMC described the time during and after KMC as relaxed, calm, happy, natural, instinctive, and safe [25–30]. Parents reported that the bonding associated with KMC felt connected, familiarizing, comforting, and logical. Mothers preferred KMC to traditional incubators because they felt closer to their babies, and it put them at ease [31].

#### Social support for caregivers

Social support referred to the perception and reality that one has assistance from other people to perform KMC. While practicing KMC, mothers and fathers did not feel supported by their families or communities [15, 25]. Mothers experienced a lack of support from healthcare workers. Some hospital staff were resistant to family participation in caring for the baby while in the hospital [32]. Healthcare workers were occasionally considered to be loud and uncaring by parents [33, 34]. Additionally, KMC was impaired when parents perceived that HCWs did not respect family privacy [35]. Fathers reported lack of support from society and frequently voiced discomfort about performing KMC because of societal norms, as many fathers felt that childcare should be the role of the mother [15, 36]. Older generations, mothers-in-law, and grandmothers in particular, did not find KMC to be an appropriate method to care for newborns [37].

In contrast, KMC uptake was promoted by societal acceptance of paternal participation in childcare, by family and community acceptance of KMC, and by the presence of engaged HCWs [38, 39]. In societies where gender roles were more equal, there were fewer barriers to fathers performing KMC [39, 40]. Paternal involvement played a large role in KMC uptake–either by division of labor or by helping the mother feel comfortable [41]. Mothers were grateful to have someone help them during KMC, such as grandmothers and sisters, who could take care of housework and help with the newborn. Within the maternity ward, peer support from other mothers who shared their KMC experiences also promoted acceptance [36, 42]. Additionally, the presence of well-trained nurses reduced maternal apprehension about practicing KMC and handling their newborn, facilitating the implementation of KMC [43].

#### Caregiver time for KMC adoption

KMC guidelines recommend continuous SSC for as long as possible until the newborn reaches a certain weight (usually 2000 g), certain age (usually 2 weeks after birth), or no longer tolerates it [4]. The lengthy time needed to provide KMC was a barrier for caregivers. KMC was difficult to perform at long intervals if the mother was depressed, lonely, or recently had a C-section [44]. Many mothers found KMC performance at home to be a burden due to other responsibilities at home or work. For example, one mother said *“Obviously I’ve got a husband and another child at home, and obviously have to cook…you have to clean and do a lot of other things, besides looking after yourself and the baby”* (Mother) [42]. Another mother noted: *‘Although I am very satisfied with the KMC method, it made me feel divided, as I was unable to be close to my other child. This must be even more complicated for those whose infant needs a longer period of hospital stay.”* (Mother) [27]. Another difficulty was commuting between home and KMC wards [17, 18, 27, 36, 39, 42, 45, 46]. Thus, the ability to practice KMC at home, rather than in a facility, promoted the uptake of KMC by allowing caregivers to attend to other chores [47]. Parents (as well as staff) noted that unlimited visitation hours enabled adoption of KMC. Furthermore, facility staff felt as though parents were less interfering when they were allowed unhindered access to their babies [48].

#### Caregiver medical concerns

Medical concerns, including the clinical condition of the mother or newborn, may also be a barrier that prevents KMC uptake. For examples, mothers in Ghana found SSC problematic because they fear that by touching the umbilical cord of the newborn it would “divide into two,” and cause pain, bleeding, or sickness [49]. Clinical consequences of KMC for mothers included fatigue, depression, and postpartum pain. Some mothers experienced discomfort sleeping upright with a newborn in KMC position [13, 39]. Postpartum pain was considered a hindrance to SSC, especially after a C-section [29, 39, 49, 50]. However, women practicing KMC thought it helped them to recover from postpartum depression [51]. Mothers seemed to be satisfied with the method and felt that it helped relieve stress [31, 52].

### Health system barriers and enablers for caregiver adoption of KMC

Adoption of KMC by caregivers generally begins in the context of the health system, and caregivers may interact with any of the core components of a health system. We found that financing and service delivery were aspects of the health system that influenced caregiver adoption of KMC.

#### Financing

First, in the case that the newborn remained in the hospital after the mother was discharged, lack of money for transportation and the distance to the hospital were often reported as the biggest challenges to KMC implementation; these were also barriers to returning to the health facility for follow up after both mother and infant were discharged but continuing KMC [53–56]. In an evaluation of a Kangaroo Care inpatient ward of a tertiary hospital in Malawi, 10% mothers whose children died reported the distance to the health facility or lack of transport money as the reason they did not go the hospital when something was wrong with their newborn; similarly, nearly 40% of mothers reported lack of transport money as the reason they did not go the hospital for their follow up clinic appointments [56]. In Kuala Lumpur, Malaysia, poor public transportation and the difficulty of returning to the hospital after restarting work were the most frequently mentioned challenges to performing skin-to-skin contact on a daily basis while the newborn was still hospitalized [55]. On the other hand, free medical service enabled parents to stay at the clinic longer as needed. Also, parents in Harare, Zimbabwe believed that KMC decreased the cost of hospital bills and assumed that it was a cheaper option than conventional incubator care or a prolonged hospital stay [15].

#### Service delivery

For caregivers, lack of privacy and KMC resources at facilities presented obstacles to KMC adoption (Appendix). Structurally, there was a lack of private space for mothers to perform KMC and a lack of space for mothers to remain in the hospital with the newborn [57, 58]. Mothers felt uncomfortable and exposed as staff continued to come in and out during KMC [25]. For example, *“There were always people around. It is harder (to be skin to skin) when there are people other people coming in and out. Private rooms will help.”* (Mother) [50]. Another mother reported: *“From seven in the morning until five in the afternoon people came in all the time. People came in to clean the room, clean the bathroom, to check on me and someone else to check on the baby. Every fifteen minutes someone different would come in. I could never relax. It was exhausting. It was stressful. I couldn’t relax.”* (Mother) [50]. Lack of resources at facilities (e.g. chairs, beds, linens, curtains, KMC wraps, etc.) was also a barrier to adoption. At one facility materials donated for KMC were put into VIP units rather than the KMC ward [54].

However, the provision of private spaces, a quiet atmosphere, and dedicated resources promoted the acceptance and uptake of KMC [34]. Privacy screens or private rooms allowed the family separation from hospital staff and other patients and offered a quieter atmosphere for the mothers to conduct KMC [59].

### Social and cultural barriers and enablers for caregiver adoption of KMC

We hypothesized that the broad social context (e.g. demographic, economic, and cultural factors) influence caregiver adoption of KMC. For example, surveys from 15 low-income countries noted that health care professionals often found that KMC was thought of as substandard or as “the poor man’s alternative” [36]. Also, caregiver adherence to traditional newborn practices was reported as a barrier to KMC [55]. Traditional early bathing behavior was seen as having numerous benefits and was identified as an ingrained behavior by studies conducted in Ghana and Bangladesh [17, 60]. One traditional birth attendant noted: *“The child needs to be bathed immediately in order to shape the head because whenever a child is delivered the head is very flat so you need to sharpen it to make it round”* [60]. A mother who had recently delivered noted that *“Babies are normally bathed shortly after birth because it will help them feel clean and healthy”* [60]. Other traditional practices, such as sleeping by a lamp and smearing the baby with oil, make uptake of KMC more difficult. In reports from Ghana and Malawi, where carrying the baby on the back was common, it seemed strange to place the baby on the front, as instructed by KMC. One woman explained: *“The back is stronger than the front and better for carrying”* [49]. In some contexts, it was considered unclean to have the mother carry the baby on her chest without a diaper [36]. Stigma surrounding having a preterm infant can be severe and can act as a barrier to continued practice of KMC.

Different approaches to gender roles, the role of parents in childcare, the role of men in the household, and the roles of other family members also influenced KMC uptake [15, 32, 36]. For example, some fathers reported feeling uncomfortable practicing KMC in public, learning how to perform KMC while other people were present, or being scrutinized by nurses [42, 61]. Additionally, in some cases, mothers and traditional birth attendants reported feeling uncomfortable with the father performing their duties [25].

## Discussion

Kangaroo mother care is a complex intervention, as defined in our conceptual framework, because user (i.e. caregiver) engagement is high and caregiver (i.e. the primary ‘adoption system’) behavior dominates our definition of ‘successful implementation’ of the intervention [8]. Thus, scale-up of this intervention around the world relies heavily on enabling caregivers to successfully adopt KMC. We found that buy-in and bonding, social support, time, and medical concerns were major themes defining the interaction between families and the KMC intervention. Furthermore, we identified financing and service delivery as barriers (and potential enablers) of KMC adoption within the context of the health system. Additionally we identified social and cultural norms that played an important role in the adoption of KMC.

Efforts to implement and scale up KMC must work to ensure a positive experience for caregivers. For example, the benefits of KMC to the newborn and caregivers must be clearly explained to everyone involved. One approach is to ensure healthcare workers present information about KMC in a standardized manner to caregivers and extended families, with attention paid to their concerns. Additionally, testimonials about the effectiveness of KMC could be given by caregivers who have successfully cared for a preterm or low birthweight baby in the past; such an approach is recommended in the Maternal and Child Health Integrated Program (MCHIP) KMC Guide [62]. Demonstrations and supervised practice can enhance caregiver confidence. Approaches to enhance newborn-caregiver bonding are needed. For example, programs might create a song about KMC to be sung to the baby, or healthcare workers could demonstrate the change in infant temperature after a period of skin to skin contact [63]. Despite efforts and ideas from programs and practitioners about how to create a positive KMC experience for caregivers, there is limited evidence about which approaches are effective.

We found that social support can enhance the uptake and duration of KMC. To enhance social support and promote positive attitudes about KMC, the Maternal and Child Health Integrated Program (MCHIP) KMC Guide recommends that programs undertake sensitization to KMC at the national, health facility, and community levels. At the community-level, recommended activities include celebrations for the ‘graduation’ of a baby from KMC or discussions about KMC through radio or other public forums [62]. Similarly, a program in Malawi asked respected grandparents to promote KMC and newborn care behavior. In the *Agogo* (the Chitumbuka word for grandparent) Program, the Ekwenedni Church of Central Africa Presbyterian (CCAP) Mission Hospital trained nearly 4000 grandparents. Subsequently, grandparents provided individual and group counseling in their respective villages, using drama, song, and poems to share key messages. An evaluation of the program concluded that grandparents were successful in promoting behavior change surrounding maternal and newborn care [64]. Encouraging family members to provide support by assisting mothers with other household responsibilities or coaching mothers how to ask for this support may also increase duration of KMC after hospital discharge. Currently, there is limited evidence about the effectiveness of such sensitization efforts.

Interactions between healthcare workers and families may either encourage or discourage caregiver adoption of KMC. This is consistent with research which demonstrated that interpersonal healthcare worker behavior is a significant contributor to patient satisfaction with maternal health services, which subsequently influences service utilization [65–67]. Healthcare workers and facilities can be supportive in their words and actions, by providing privacy for the family as they learn KMC and by ensuring unlimited visitation hours so that KMC can happen without time or schedule constraints. Health system concerns regarding financing travel, food, lodging, etc. may be partially alleviated by ensuring early discharge of mother and infant from the hospital (which should always be included as a component of KMC). Additionally, KMC programs may consider ways to reduce hospital charges or provide transportation vouchers for families of infants with longer-than-average stays. For example, some programs in Colombia maintain social funds to provide financial support to families who must travel to a health facility in the period of close follow up after the newborn is discharged from the hospital (*personal communication with Nathalie Charpak, Director Fundación Canguro*). Automated cash transfers using cell phone technology, might be a method to reduce financial barriers. Transportation and time costs may also be addressed by offering home visits by community health workers for infant follow up. Further studies are needed to generate evidence regarding the feasibility and effectiveness of such an approach.

It is important to acknowledge that mothers, fathers, and families are adopting KMC within a broader social context. Several studies specifically noted that there may be stigma associated with having a preterm infant or around male involvement in child care, which present barriers to KMC uptake. Divisions of labor and space by gender have been found to be barriers to male participation in newborn care, in general. However, as Dumbaugh *et al* note, inclusion of men in newborn care must be done in a way that is empowering for women [68]. To address the reluctance of fathers to engage in childcare, fathers successfully engaging in SSC might become peer-mentors or demonstrators for other families. The intervention name “Kangaroo Mother Care” might also be changed so that it does not directly imply the behavior is performed only by the mother. Additional research about how to encourage paternal involvement and reduce stigma surrounding these childcare strategies must be a part of KMC scale up in any context.

### Strengths & limitations

The primary strength of this research is that it draws on the rich body of qualitative research that can help policy-makers and public health professionals to understand the complex context in which this intervention is implemented. However, our conclusions are limited by the existing body of evidence. There has been less research conducted in Southeast Asia and sub-Saharan Africa where KMC has the potential make the greatest impact. Furthermore, nearly half of the studies were conducted in urban settings with low neonatal mortality (<15 per 1000 live births). Additional research is needed in the places where KMC has the potential for the highest impact and should be geared towards understanding the needs of caregivers of preterm and low birthweight infants. Future research should also investigate ways to generate demand for KMC services.

## Conclusion

We found that lack of buy-in, poor social support, lack of time at the hospital or at home, and medical concerns about the mother or infant were barriers to caregiver adoption of KMC. Furthermore, we identified barriers and enablers of KMC adoption by families within the context of the health system and the broader social context. Future efforts to integrate KMC into local, regional, or national health systems must make efforts to identify and reduce barriers and promote enablers for successful caregiver adoption of KMC. Ultimately, KMC programs must ensure that the KMC experience is a valuable and positive experience from the caregiver perspective.

## Appendix

**Table 3:** Studies included in the systematic review of barriers and enablers (*n* = 98)

Author	Year	Title	Country	Rural or Urban	Study design	Sample Size	Newborn Description
Bergh	2014	Implementing facility-based kangaroo mother care services: lessons from a multi-country study in Africa	Malawi, Mali, Rwanda, and Uganda	Urban	Facility evaluation, Focus group/interview	39 facilities	N/A
Morelius	2015	Neonatal nurses’ beliefs about almost continuous parent-infant skin-to-skin contact in neonatal intensive care	Sweden	Urban	Survey	129 nurses	All newborns
Nahidi	2014	Opinions of the midwives about enabling factors of skin-to-skin contact immediately after birth: A descriptive study	Iran	Urban	Questionnaire	292 midwives	N/A
Zwedberg	2015	Midwives’ experiences with mother-infant skin-to-skin contact after a caesarean section: ‘fighting an uphill battle’	Sweden	Urban	Focus group/interview	8 midwives	N/A
Abul-Fadl	2012	Evaluation of mothers’ knowledge, attitudes, and practice towards the ten steps to successful breastfeeding in Egypt	Egypt	Mixed	Pop based surveillance, facility evaluation	1052 Mothers	All ages
Alves	2007	Early weaning in premature babies participants of the Kangaroo Mother Care	Brazil	Mixed	Chart review, Focus group/interview	33 Dyads	Premature; N/A cutoff
Araújo	2010	Mother Kangaroo Method: an investigation about the domestic practice	Brazil	Urban	Focus group/interview	30 Parents	Premature, ≥2000 g
Arivabene	2010	Kangaroo mother method: Mothers’ experiences and contributions to nursing	Brazil	Urban	Focus group/interview	13 Mothers	N/A
Bazzano	2012	Introducing home based skin-to-skin care for low birth weight newborns: a pilot approach to education and counseling in Ghana	Ghana	Rural	Focus group/interview	9 Mother, 23 HCWs	LBW; N/A cutoff
Bergh	2008	Scaling up kangaroo mother care in South Africa: ‘on-site’ versus ‘off-site’ educational facilitation	South Africa	Mixed	RCT	36 Facilities	N/A
Bergh	2012	Progress in the implementation of kangaroo mother care in 10 hospitals in Indonesia	Indonesia	Urban	Facility evaluation	10 Facilities	N/A
Bergh	2012	Translating research findings into practice—the implementation of kangaroo mother care in Ghana	Ghana	N/A	Pop based surveillance, facility evaluation	38 Facilities	N/A
Bergh	2013	Progress with the implementation of kangaroo mother care in four regions in Ghana	Ghana	N/A	Facility evaluation	38 Facilities	N/A
Bergh	2007	Retrospective Evaluation of Kangaroo Mother Care Practices in Malawian Hospitals	Malawi, South Africa	N/A	Facility evaluation	6 Facilities	N/A
Bergh	2009	Scaling up Kangaroo Mother Care in Ghana	Ghana	N/A	Facility evaluation	4 Regions (out of 10)	N/A
Bergh	2012	Evaluation of Kangaroo Mother Care Services in Malawi	Malawi	N/A	Facility evaluation	14 facilities	N/A
Bergh	2012	Evaluation of Kangaroo Mother Care Services in Mali	Mali	N/A	Facility evaluation	7 Facilities	N/A
Bergh	2012	Evaluation of Kangaroo Mother Care Services in Rwanda	Rwanda	N/A	Facility evaluation	7 Facilities	N/A
Bergh	2012	Evaluation of Kangaroo Mother Care Services in Uganda	Uganda	N/A	Facility evaluation	11 Facilities	N/A
Blencowe	2005	Setting up Kangaroo Mother Care at Queen Elizabeth Hospital, Blantyre–A practical approach	Malawi	Urban	Facility evaluation	1 Facility	<2000 g
Blencowe	2009	Safety, effectiveness and barriers to follow-up using an ‘early discharge’ Kangaroo Care policy in a resource poor setting	Malawi	Urban	Prospective cohort	272 Newborns	<2000 g
Blomqvist	2011	Swedish mothers’ experience of continuous Kangaroo Mother Care	Sweden	Urban	Focus group/interview	23 Dyads	All ages
Blomqvist	2013	Provision of Kangaroo Mother Care: supportive factors and barriers perceived by parents	Sweden	N/A	Focus group/interview	76 Mother, 74 Fathers	28–33 weeks, 740–2920 g
Boo	2007	Short duration of skin-to-skin contact: Effects on growth and breastfeeding	Malaysia	Urban	RCT	126 Dyads	<1501 g
Brimdyr	2012	A Realistic Evaluation of Two Training Programs on Implementing Skin-to-Skin as a Standard of Care	Egypt	N/A	Focus group/interview	40 Nurses and HCWs	N/A
Calais	2010	Skin-to-skin contact of full term infants: an explorative study of promoting and hindering factors in two Nordic childbirth settings	Sweden, Norway	Urban	Focus group/interview	117 Mother, 107 Fathers	Full term
Castiblanco López	2011	Vision of mothers in care of premature babies at home	Colombia	Urban	Focus group/interview	8 Mothers	<36 weeks, 2320 g
Charpak	2006	Resistance to implementing Kangaroo Mother Care in developing countries, and proposed solutions	15 developing countries	Mixed	Focus group/interview	17 KMC co-ordinators, 15 Facilities	N/A
Chia	2006	The attitudes and practices of neonatal nurses in the use of kangaroo care	Australia	Urban	Focus group/interview	34 Nurses	N/A
Chisenga	2015	Kangaroo Mother Care: A review of mothers’ experiences at Bwaila hospital and Zomba Central hospital (Malawi)	Malawi	Urban	Focus group/interview	113 mothers	N/A
Colameo	2006	Kangaroo Mother Care in public hospitals in the State of Sao Paulo, Brazil: an analysis of the implementation process	Brazil	Mixed	Cross sectional	28 Facilities	LBW; N/A cutoff
Cooper	2014	Close to me: enhancing kangaroo care practice for NICU staff and parents	USA	Mixed	Pre-post	48 nurses and 101 parents	N/A
Crenshaw	2012	Use of a video-ethnographic intervention (PRECESS Immersion Method) to improve skin-to-skin care and breastfeeding rates	USA	N/A	Descriptive	261 Dyads	Full term
Dalal	2014	A cross-sectional study on knowledge and attitude regarding kangaroo mother care practice among health care providers in Ahmedabad district	India	Mixed	Cross sectional	145 HCPs	N/A
Dalbye	2011	Mothers’ experiences of skin-to-skin care of healthy full-term newborns–A phenomenology study	Sweden, Norway	Urban	Focus group/interview	20 Mothers	Full term
Darmstadt	2006	Introduction of community-based skin-to-skin care in rural Uttar Pradesh, India	India	Rural	Intervention	2063 Mothers	All ages
Duarte	2001	Kangaroo mother care: experience report	Brazil	Urban	Focus group/interview	1 Mother	Premature; N/A cutoff
Eichel	2001	Kangaroo care: Expanding our practice to critically iII neonates	USA	Urban	Facility evaluation	1 Facility	N/A
Engler	2002	Kangaroo care: national survey of practice, knowledge, barriers, and perceptions	USA	Mixed	Facility evaluation	537 Facilities	N/A
Ferrarello	2014	Barriers to skin-to-skin care during the postpartum stay	USA	Urban	Focus group/interview	15 Mother, 14 Nurses	N/A
Flynn	2010	Neonatal nurses’ knowledge and beliefs regarding kangaroo care with preterm infants in an Irish neonatal unit	Ireland	Urban	Focus group/interview	62 HCWs	N/A
Furlan	2003	Perception of parents in experiencing the kangaroo mother method	Brazil	Urban	Focus group/interview	10 Parents	Premature; N/A cutoff
Gontijo	2010	Evaluation of implementation of humanized care to low weight newborns: the Kangaroo Method	Brazil	Mixed	Facility evaluation	293 Facilities	N/A
Gonya	2013	Factors associated with maternal visitation and participation in skin-to-skin care in an all referral level IIIc NICU	USA	Urban	Focus group/interview	32 Mothers	<27 weeks
Haxton	2012	Implementing skin-to-skin contact at birth using the Iowa model: applying evidence to practice	USA	Urban	Intervention, qualitative	30 Mothers	All ages
Heinemann	2013	Factors affecting parents’ presence with their extremely preterm infants in a neonatal intensive care room	Sweden	N/A	Focus group/interview	7 Mother, 6 Fathers	<27 weeks
Hendricks-Munoz	2013	Maternal and Neonatal Nurse Perceived Value of Kangaroo Mother Care and Maternal Care Partnership in the Neonatal Intensive Care Unit	USA	Urban	Focus group/interview	143 Mother, 42 HCWs	<34 weeks
Hendricks-Munoz	2014	A Neonatal Nurse Training Program in Kangaroo Mother Care (KMC) Decreases Barriers to KMC Utilization in the NICU	USA	Urban	Prospective cohort	30 nurses	N/A
Hennig	2006	Health professional’s knowledge and practices about KMC	Brazil	Mixed	Cross sectional	148 Doctors and nurses, 11 Facilities	LBW; N/A cutoff
Higman	2015	Assessing clinicians’ knowledge and confidence to perform kangaroo care and positive touch in a tertiary neonatal unit in England using the Neonatal Unit Clinician Assessment Tool (NUCAT)	England	Urban	Focus group/interview	6 nurses and 51 clinicians	N/A
Hill	2010	Keeping newborns warm: beliefs, practices and potential for behavior change in rural Ghana	Ghana	Mixed	Focus group/interview	635 Mother, 14 Villages	All ages
Hunter	2014	Newborn care practices in rural Bangladesh: Implications for the adaptation of kangaroo mother care for community-based interventions	Bangladesh	Rural	Focus group/interview	121 participants	N/A
Ibe	2004	A comparison of kangaroo mother care and conventional incubator care for thermal regulation of infants < 2000 g in Nigeria using continuous ambulatory temperature monitoring	Nigeria	Urban	Crossover	13 Newborns, 11 Mothers and female relatives	1200–1999 g
Johnson	2007	Factors influencing implementation of kangaroo holding in a Special Care Nursery	USA	Peri-urban/Slum	Focus group/interview	17 Nurses	N/A
Johnston	2011	Paternal vs. maternal kangaroo care for procedural pain in preterm neonates: a randomized crossover trial	Canada	N/A	RCT crossover	62 Newborns	28–36 weeks
Kambarami	2002	Caregivers’ perceptions and experiences of ‘kangaroo care’ in a developing country	Zimbabwe	Urban	Focus group/interview	40–48 mothers (4 focus groups with 10–12 participants per group)	LBW: N/A cutoff
Keshavarz	2010	Effects of kangaroo contact on some physiological parameters in term neonates and pain score in mothers with cesarean section	Iran	Urban	RCT	160 Dyads	Full term
Kymre	2013	Balancing preterm infants’ developmental needs with parents’ readiness for skin-to-skin care: A phenomenological study	Sweden, Norway, Denmark	N/A	Focus group/interview	18 Nurses	N/A
Lee	2012	Clinician perspectives on barriers to and opportunities for skin-to-skin contact for premature infants in neonatal intensive care units	USA	Mixed	Focus group/interview	69 HCPs, 11 Facilities	N/A
Legault M	1995	Comparison of kangaroo and traditional methods of removing preterm infants from incubators	Canada	Urban	RCT, pre-post, cross over	61 Dyads	Premature: N/A cutoff 1000–1800 g
Lemmen	2013	Kangaroo care in a neonatal context: parents’ experiences of information and communication of nurse-parents	Sweden	N/A	Focus group/interview	12 Families	24–35 weeks
Leonard	2008	Parents’ Lived Experience of Providing Kangaroo Care to their Preterm Infants	South Africa	Urban	Focus group/interview	6 Parents	Premature: N/A cutoff
Lincetto	1998	Impact of season and discharge weight on complications and growth of Kangaroo Mother Care treated low birth weight infants in Mozambique	Mozambique	Urban	Prospective cohort	246 Newborns	<2000 g
Maastrup	2012	Breastfeeding Support in Neonatal Intensive Care: A National Survey	Denmark	N/A	Facility evaluation	19 Facilities	N/A
Mallet	2007	[Skin to skin contact in neonatal care: knowledge and expectations of health professionals in 2 neonatal intensive care units]	France	N/A	Focus group/interview	121 Doctors and paramedical staff	N/A
Martins	2008	Living in the other side of kangaroo method: the mother’s experience	Brazil	Urban	Focus group/interview	5 Mothers	N/A
McMaster	2000	Kangaroo care in Port Moresby, Papua New Guinea	Papua New Guinea	Urban	Chart review, facility evaluation	109 Newborns	<1500 g
Moreira	2009	Kangaroo mother program and the relationship mother-baby: qualitative research in a public maternity of Betim city	Brazil	Urban	Focus group/interview	8 Mothers	30–32 weeks, <2000 g
Namazzi	2015	Strengthening health facilities for maternal and newborn care: experiences from rural eastern Uganda	Uganda	Rural	RCT	20 health facilities	All newborns
Neu	1999	Parents’ perception of skin-to-skin care with their preterm infants requiring assisted ventilation	N/A	Urban	Focus group/interview	8 Mothers, 1 Father	Premature; N/A cutoff
Nguah	2011	Perception and practice of Kangaroo Mother Care after discharge from hospital in Kumasi, Ghana: a longitudinal study	Ghana	Urban	Prospective cohort	195 Dyads	1000–2000 g
Niela-Vilen	2013	Early physical contact between a mother and her NICU-infant in two university hospitals in Finland	Finland	Urban	Prospective cohort, qualitative	170 Mothers, 381 Staff	all NICU newborns
Nyqvist	2008	Application of the baby friendly hospital initiative to neonatal care: suggestions by Swedish mothers of very preterm infants	Sweden	N/A	Focus group/interview	13 Mothers	<32 weeks
Parmar	2009	Experience with Kangaroo mother care in a neonatal intensive care unit (NICU) in Chandigarh, India	India	Urban	Retrospective cohort	135 Newborns	26–37 weeks, 550–2500 g
Priya	2004	Kangaroo care for low birth weight babies	India	N/A	Crossover	30 Dyads	LBW; N/A cutoff
Quasem	2003	Adaptation of kangaroo mother care for community-based application	Bangladesh	Urban	Focus group/interview	35 Mothers	All ages
Ramanathan	2001	Kangaroo Mother Care in very low birth weight infants	India	N/A	RCT	28 Newborns	<1500 g
Roller	2005	Getting to know you: mothers’ experiences of kangaroo care	USA	N/A	Focus group/interview	10 Mothers	32–37 weeks
Sá	2010	Interpersonal relationships between professionals and mothers of premature from Kangaroo-Unit	Brazil	Urban	Focus group/interview	10 Mothers, 7 HCPs	Premature; N/A cutoff
Sacks	2013	Neonatal care in the home in northern rural Honduras: a qualitative study of the role of traditional birth attendants	Honduras	Rural	Focus group/interview	48–72 TBAs (6 focus groups with 8–12 participants per group)	N/A
Santos	2013	Maternal perception of the skin to skin contact with premature infants through the kangaroo position	Brazil	Urban	Focus group/interview	12 Mothers	Premature, LBW; N/A cutoff
Shamba	2014	Thermal care for newborn babies in rural southern Tanzania: a mixed-method study of barriers, facilitators and potential for behaviour change	Tanzania	Mixed	Focus group/interview	57 mothers and 14 TBAs	N/A
Silva	2008	Perceptions and neonatal care behavior of women in a Kangaroo-mother Care Program	Brazil	Urban	Focus group/interview	5 Dyads	Premature: N/A cutoff, <1000–1550 g
Silva	2014	Conhecimento de técnicos de enfermagem sobre o método canguru na unidade neonatal; Nursing technicians? knowledge of the Kangaroo-Mother Care method in the neonatal unit; Conocimiento de técnicos de enfermería sobre el método Canguro en la unidad neonatal	Brazil	Urban	Focus group/interview	20 nursing technicians	N/A
Sinha	2014	Newborn care practices and home-based postnatal newborn care programme–Mewat, Haryana, India, 2013	India	Rural	Focus group/interview	320 mothers, 61 Accredited Social Health Activists, 19 home visits	N/A
Sloan	2008	Community-based kangaroo mother care to prevent neonatal and infant mortality: a randomized, controlled cluster trial	Bangladesh	Rural	Cluster RCT	39,888 Mothers	All ages
Solomons	2012	Knowledge and attitudes of nursing staff and mothers towards kangaroo mother care in the eastern sub-district of Cape Town	South Africa	Urban	Cross sectional	30 Mothers, 15 Nurses	<2500 g
Stikes	2013	Applying the plan-do-study-act model to increase the use of kangaroo care	USA	Urban	Focus group/interview	56 Nurses	N/A
Strand	2014	Kangaroo mother care in the neonatal intensive care unit: staff attitudes and beliefs and opportunities for parents	Sweden	N/A	Facility evaluation	126 Staff	N/A
Tessier	1998	Kangaroo mother care and the bonding hypothesis	Colombia	Urban	RCT	488 Newborns	<2001 g
Toma	2003	Kangaroo Mother Care: the role of health care services and family networks in a successful program	Brazil	Urban	Focus group/interview	14 Mothers, 7 Fathers	Premature: N/A cutoff, 1150–2300 g
Toma	2007	Maternal perception of low birth weight babies before and following the implementation of the Kangaroo Mother Care in a public hospital, in the city of São Paulo, Brazil	Brazil	Urban	Focus group/interview	41 Mothers	<2000 g
Undefined author: Save the Children	2011	Scaling Up Kangaroo Mother Care, Report of Country Survey Findings	Ethiopia, Malawi, Mali, Mozambique, Nigeria, Tanzania, Uganda, Bolivia, Indonesia, Nepal, Vietnam	N/A	Facility evaluation	12 Countries	N/A
Vesel	2013	Promoting skin-to-skin care for low birth weight babies: findings from the Ghana Newhints cluster-randomized trial	Ghana	Rural	Cluster RCT	98 Zones	All ages
Waiswa	2010	‘I never thought that this baby would survive; I thought that it would die any time’: perceptions and care for preterm babies in eastern Uganda	Uganda	Rural	Focus group/interview	30 HCW and mothers, 16 Facilities	Premature; N/A cutoff
Waiswa	2015	Effect of the Uganda Newborn Study on care-seeking and care practices: a cluster-randomised controlled trial	Uganda	Rural	Cluster RCT	395 women	All newborns
Wobi	2010	Report on the Kangaroo Mother Care Project at Komfo Anokye Teaching Hospital in Kumasi, Ghana	Ghana	Urban	Facility evaluation	2 Facilities	N/A
Zhang	2014	Evidence utilization project: implementation of kangaroo care at neonatal ICU	Singapore	Urban	Facility evaluation	1 ICU	<34 weeks; < 1500 g

## Abbreviations

KMC: Kangaroo mother care
SSC: Skin-to-skin contact
